# Application of the NanoString nCounter System as an Alternative Method to Investigate Molecular Mechanisms Involved in Host Plant Responses to *Plasmodiophora brassicae*

**DOI:** 10.3390/ijms232415581

**Published:** 2022-12-08

**Authors:** Qinqin Zhou, Leonardo Galindo-González, Sheau-Fang Hwang, Stephen E. Strelkov

**Affiliations:** 1 Department of Agricultural, Food and Nutritional Science, University of Alberta, Edmonton, AB T6G 2P5, Canada; 2 Molecular Identification Research Laboratory, Canadian Food Inspection Agency, Ottawa, ON K2H 8P9, Canada

**Keywords:** *Plasmodiophora brassicae*, clubroot, NanoString nCounter system, qPCR, RNA-seq, gene expression

## Abstract

Clubroot, caused by the soilborne pathogen *Plasmodiophora brassicae*, is an important disease of canola (*Brassica napus*) and other crucifers. The recent application of RNA sequencing (RNA-seq) technologies to study *P. brassicae*–host interactions has generated large amounts of gene expression data, improving knowledge of the molecular mechanisms of pathogenesis and host resistance. Quantitative PCR (qPCR) analysis has been widely applied to examine the expression of a limited number of genes and to validate the results of RNA-seq studies, but may not be ideal for analyzing larger suites of target genes or increased sample numbers. Moreover, the need for intermediate steps such as cDNA synthesis may introduce variability that could affect the accuracy of the data generated by qPCR. Here, we report the validation of gene expression data from a previous RNA-seq study of clubroot using the NanoString nCounter System, which achieves efficient gene expression quantification in a fast and simple manner. We first confirm the robustness of the NanoString system by comparing the results with those generated by qPCR and RNA-seq and then discuss the importance of some candidate genes for resistance or susceptibility to *P. brassicae* in the host. The results show that the expression of genes measured using NanoString have a high correlation with the values obtained using the other two technologies, with *R* > 0.90 and *p* < 0.01, and the same expression patterns for most genes. The three methods (qPCR, RNA-seq, and NanoString) were also compared in terms of laboratory procedures, time, and cost. We propose that the NanoString nCounter System is a robust, sensitive, highly reproducible, and simple technology for gene expression analysis. NanoString could become a common alternative to qPCR to validate RNA-seq data or to create panels of genes for use as markers of resistance/susceptibility when plants are challenged with different *P. brassicae* pathotypes.

## 1. Introduction

Clubroot, caused by the obligate parasite *Plasmodiophora brassicae*, is a devastating soilborne disease of *Brassica* species worldwide [1]. Susceptible hosts infected by the pathogen develop large galls on the roots that interrupt nutrient and water uptake, resulting in losses in crop yield estimated at 10–15% globally [2]. In Canada, clubroot disease is a major concern for canola (oilseed rape; *Brassica napus* L.) production, which is worth 26.7 billion CAD annually to the national economy [3]. The cultivation of clubroot-resistant (CR) cultivars is the most common and effective disease management strategy in Canada [4,5]. However, the selection pressure caused by continued planting of CR cultivars has led to the emergence of new *P. brassicae* pathotypes that can overcome host resistance [6]. To date, over 35 *P. brassicae* pathotypes have been identified in Canada, based on their classifications on the Canadian clubroot differential (CCD) set [7,8,9,10]. Given the continuing spread of clubroot in western Canada and the increasing prevalence of new virulent pathotypes of *P. brassicae* [7,10], it is important to speed up clubroot resistance breeding efforts to achieve the long-term and sustainable management of this disease. To achieve this goal, research is underway to identify new clubroot resistance genes and to improve the understanding of *P. brassicae*–host interactions [11,12,13,14].

Gene expression analysis, which investigates gene regulation at the transcriptional level, has been increasingly applied to study host responses to *P. brassicae*, serving to identify genes that may be involved in the interaction or contribute to resistance [11,12,15]. In the last decade, RNA sequencing (RNA-seq) has been used extensively in studies of the host response to clubroot [12] due to its advantages, such as detecting new transcripts, a large dynamic range to quantify gene expression levels, and cost effectiveness in detecting all the expressed genes [16]. In this context, quantitative PCR (qPCR) is mainly used to validate results generated by RNA-seq [11,17]. In addition, interest has increased in studying the regulation of genes in specific gene families or pathways that may be involved in mediating the host–*P. brassicae* interaction, with qPCR analysis often applied to achieve this goal. For example, qPCR was used to study the Mitogen-activated Protein Kinase (*MPK*) and *MPK* kinase gene families [18], the Myrosinase and Myrosinase-Binding Protein-like gene families [19], and the chitinase [20] and SWEET gene families [21]. Auxin signaling [22], cytokinin signaling [23], starch metabolism [24], and hormone signaling and phenylpropanoid-related genes [25] have also been evaluated by qPCR. However, as the scale of gene expression studies increases, with more targeted genes and larger sample sizes (e.g., cultivars with different levels of susceptibility, different tissues, and different infection stages), performing qPCR can be time-consuming, laborious, and less cost-effective. Moreover, while RNA-seq is time- and cost-effective for high-throughput gene expression studies, it may not be cost-effective when studying only a limited number of genes or subsets of genes found to be important in other transcriptomic studies. Therefore, a more efficient and cost-effective method will be helpful in speeding up studies on the molecular mechanisms involved in host–*P. brassicae* interactions.

The NanoString nCounter System (NanoString Technologies, Seattle, WA, USA) is a hybridization-based technology allowing digital quantification of up to 800 multiplexed target genes using color-coded molecular barcodes and single-molecule imaging [26]. This technology may aid in generating large panels to test important genes across different conditions without the need of generating full transcriptomes, as well as validating RNA-seq data. In addition, the NanoString nCounter System is simpler and faster than the other two technologies in terms of sample preparation and processing, as it does not require gene amplification, reverse transcription, or library preparation steps [27]. Moreover, it prevents errors that could be introduced during gene amplification or cDNA synthesis. NanoString technology has been widely applied in studies of human disease where expression profiling for large numbers of genes or samples was conducted. For instance, 231 genes were investigated in 34 patients with gastrointestinal stromal tumors [28], 50 genes were tested in over 500 patients with breast cancer [29], and 477 genes were assessed in patients with metastatic gastric cancer [30]. Recently, the NanoString nCounter System has also been used to study gene regulation in plant–pathogen interactions. In a previous study, the NanoString nCounter System was used to measure the expression levels of seven defense marker genes in kiwifruit with 16 combinations of treatments and time-points to determine the importance of the SA pathway in plant defense when challenged with *Pseudomonas syringae* pv. *actinidiae* [31]. Similarly, the technology was applied to measure expression levels of 46 genes in tomato with 24 combinations of treatments and time-points to improve the understanding of the host’s response to different *Potato virus Y* strains [32]. In addition, this technology has been used to validate expression levels from RNA-seq data in *P. brassicae* infecting *B. napus*, showing agreement between the two technologies [33].

To test the feasibility and robustness of the NanoString nCounter System for study and validation of the host response to clubroot, we applied this technology to examine the expression levels of 29 genes in clubroot-resistant and susceptible rutabaga (*B. napus* subsp. *rapifera* Metzg.) cultivars in response to *P. brassicae* pathotype 3A using samples collected from a previous transcriptomics study [34]. These genes had been validated by qPCR analysis or were associated with the host response to clubroot disease. The results generated using the NanoString nCounter System were highly correlated with those from qPCR and RNA-seq, suggesting that the NanoString nCounter System can serve as an alternative method to validate RNA-seq data and may enhance knowledge of the host response to clubroot via the study of the expression of larger numbers of target genes.

## 2. Results

To ensure the accuracy of differential gene expression analysis, the stability of selected reference genes was evaluated by analyzing the output from the NanoString nCounter System. Specifically, the raw count values of the four reference genes were normalized by positive controls using nSolver software (NanoString Technologies); the normalized values were log_2_ transformed and analyzed using BestKeeper [35]. The results indicated that expression of these genes was stable (SD < 1) and significantly correlated (0.66 < *R* < 0.95, *p* < 0.01), similar to the results obtained by Zhou et al. [34] using qPCR analysis (Supplementary Tables S2 and S3). In addition, the Pearson correlations of the log_2_ counts of the four reference genes between each sample were plotted, confirming a high correlation (*R* > 0.9) across samples (Supplementary Figure S1). Therefore, these four genes were stable and could be used to normalize the expression of target genes.

**Figure 1:**
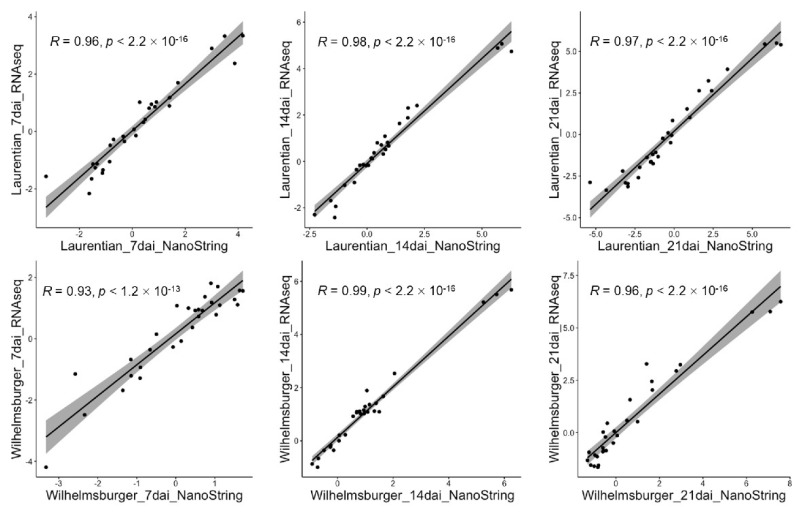
Correlation of expression of 29 genes based on log2 fold change changes, as determined using the NanoString nCounter System vs. RNA-seq analysis. The *R*-values indicate the correlation coefficient between the two methods in each host and time-point, and the *p*-values indicate the significance level of the *t*-test.

Twenty-nine target genes were tested with the NanoString nCounter System and analyzed using nSolver (NanoString Technologies). The results showed that the detected expression levels were significantly correlated (*R* > 0.90 and *p* < 0.01) with the results from the RNA-seq in all treatments (Figure 1). Among these genes, eight had also been validated using qPCR (Table S1) [34]. The expression data for the eight genes was similar whether generated with the NanoString nCounter System or by RNA-seq or qPCR (Table 1).

## 3. Discussion

### 3.1. Robustness of the NanoString nCounter System

In this study, we used the NanoString nCounter System to validate the expression of 29 genes that also had been quantified by RNA-seq and/or qPCR in an earlier transcriptomic analysis [34] of the rutabagas “Laurentian” and “Wilhelmsburger” in response to inoculation with *P. brassicae* pathotype 3A. In that earlier analysis, we had identified many genes that may play important roles in host defense against *P. brassicae*, including genes involved in the regulation of plant defense hormones and genes showing opposite regulation patterns in the resistant and susceptible cultivars [34]. Here, we validated the expression of some of the genes that had been discussed by Zhou et al. (2020) [34], including those regulating salicylic acid (SA)-mediated responses (*CBP60G*, *WRKY53*, *WRKY46*, *ATL31*, and *BSMT1*), jasmonic acid (JA)-mediated responses (*AOS*, *FAD7*, *CYP94C1*, and *CYP94B1*), a gene encoding a TIR-NBS-LRR class family protein (BnaA03g29300D), a gene encoding thylakoidal processing peptidase (*PLSP2A*), genes involved in auxin and ethylene signaling (*CYP83A1* and *ERF104*), and a gene involved in effector-triggered immunity (*MYB15*). In addition, we validated changes in the expression of genes that may be associated with clubroot resistance, including those that have been studied in the host’s response to *P. brassicae* or other pathogens (Table S1). We also tested genes that had been validated by qPCR [34] to compare the three technologies (the NanoString nCounter System, qPCR, and RNA-seq). The results of the correlation tests demonstrated that the NanoString platform was a robust tool for studying changes in gene expression in the hosts following challenge by *P. brassicae* (Figure 1 and Table 1).

**Figure 2:**
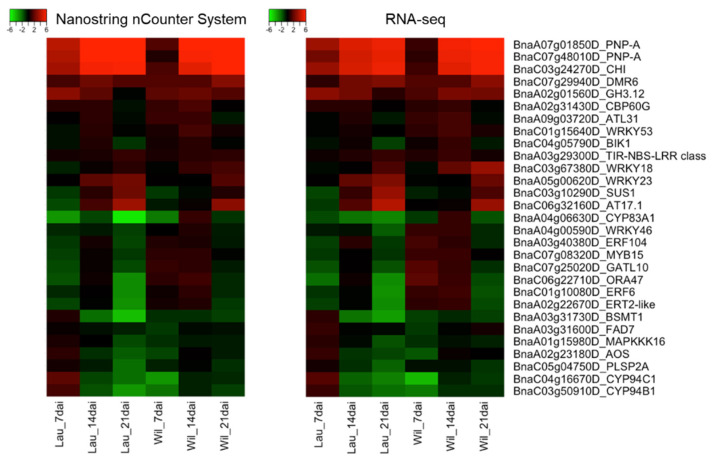
Heatmaps of 29 selected genes in a resistant rutabaga “Wilhelmsburger” (Wil) and a susceptible rutabaga “Laurentian” (Lau) in response to *Plasmodiophora brassicae* from 7 to 21 days after inoculation (dai). Results are based on log_2_ fold changes, as determined using the NanoString nCounter System and RNA-seq analysis.

In addition to the correlation tests, we constructed heatmaps of gene expression changes, as determined using the NanoString nCounter System vs. RNA-seq (Figure 2), to compare these two methods further as tools to study the expression of individual genes. The results indicated that most genes showed consistent trends in regulation when analyzed with either of the two methods, although the degree of up- or downregulation was sometimes different. Nevertheless, in most cases, the variability in the results generated with the two methods would not affect the data interpretation. For example, in the RNA-seq study, SA-mediated defense was suggested to be activated in both the resistant and susceptible cultivars in response to *P. brassicae*, but may have been stronger in the resistant cultivar because several genes that regulate SA levels were differentially regulated in the two hosts [34]. When we compared the expression of the gene encoding BSMT1, which regulates SA methylation [36], the results generated with both methods indicated upregulation in the susceptible cultivar and downregulation in the resistant cultivar at 7 dai, although this difference seemed greater in the RNA-seq data (Figure 2). In a few cases, the variability in the results generated with the two methods could affect the data interpretation. For example, *FAD7*, which regulates both the JA and SA pathways [37], was suggested as an important candidate susceptibility factor by Zhou et al. (2020) [34] because a transcript matching this gene was downregulated in the resistant cultivar but upregulated in the susceptible cultivar (log_2_ fold change = 1.02) at 7 dai. However, the data generated with the NanoString nCounter System indicated that the upregulation of this gene in the susceptible cultivar was very weak (log_2_ fold change = 0.28) (Figure 2). With this information, we may need to reconsider if this gene is indeed a potential marker of the host response to *P. brassicae*.

### 3.2. Additional Genes That May Be Important in Host Resistance to P. brassicae

To identify additional genes that could be included in future expression studies with different host genotype × *P. brassicae* pathotype combinations, we validated the regulation of some genes that may be important based on previous studies, but which were not discussed by Zhou et al. [34]. For example, the transcriptomic profiling of the *B. napus* cultivars “Laurentian” (resistant) and “Brutor” (susceptible) in response to another *P. brassicae* pathotype 5X [38] indicated the importance of SA-mediated immunity in the host response to *P. brassicae*. Therefore, we selected several genes that were upregulated by both Zhou et al. [34] and Galindo-González et al. [38] as targets for the NanoString assay. These included a plant natriuretic peptide A (*PNP-A*), gretchen hagen 3.12 (*GH3.12*, also known as *PBS3*), downy mildew resistant 6 (*DMR6*), and a putative chitinase (*CHI*). The results showed that the expression of these genes, as determined with the NanoString nCounter System, was consistent with the results from RNA-seq analysis. The only exception was *GH3.12*, which was found to be slightly upregulated (log_2_ fold change = 0.85) in the susceptible cultivar at 21 dai based on RNA-seq analysis but was not upregulated based on the NanoString technology (log_2_ fold change = −0.1) (Figure 2 and Table S1). *GH3.12*, belonging to a GH3 acyl adenylase-family enzyme, is involved in defense responses to pathogens by promoting SA biosynthesis and metabolism [39,40]. Any differences in the results generated with the NanoString nCounter System vs. RNA-seq did not affect the interpretation of the significance of this gene in the host response to clubroot. The expression of *GH3.12* at other time-points or in the resistant cultivar, as determined with the NanoString nCounter System, was consistent with the results of Galindo-González et al. [38] and Zhou et al. [34]; transcripts were upregulated in the resistant host throughout the time-course (7, 14, and 21 dai) but only at 7 dai or 7 and 14 dai in the susceptible host. *PNP-A* encodes a pathogenesis-related protein. This gene is controlled specifically by WRKY70 and is positively involved in the SA-mediated systemic acquired resistance pathway [41]. The results obtained with the NanoString nCounter System supported the same regulation pattern of *PNP-A* in the four host–pathotype combinations as reported by Zhou et al. [34] and Galindo-González et al. [38], where transcripts for *PNP-A* showed strong upregulation in both the resistant and susceptible hosts at 7, 14, and 21 dai, particularly at the latter two time-points. In these two transcriptomic studies, multiple transcripts for *WRKY70* were upregulated in both resistant and susceptible hosts throughout the time-points, suggesting *WRKY70*-*PNP-A*-SA-related immune pathways in host defense to *P. brassicae*. *DMR6*, which encodes a putative 2OG-Fe (II) oxygenase, is significantly induced by pathogen or SA treatment and regulates SA homoeostasis by hydroxylating SA into 2,5-DHBA [42]. It has been a promising target for gene editing as a susceptibility factor to increase plant resistance to broad-spectrum diseases [43,44,45]. Multiple *B. napus* transcripts for *DMR6* were upregulated at different time-points in response to *P. brassicae* pathotypes 5X and 3A [34,38], including the transcript for BnaC07g29940D, which was validated with the NanoString assay (Figure 2). In Arabidopsis accession Bur-0 (172AV), *DMR6* was upregulated during the incompatible interaction (treated with *P. brassicae* isolate eH) but was not differently regulated in the compatible interaction (treated with *P. brassicae* isolate e_2_) [46]. Therefore, it would be interesting to study the roles of *DMR6* in the host response to *P. brassicae* further to confirm if the regulation of this gene would affect host resistance to *P. brassicae*. Overall, the upregulation of these genes supports the importance of SA in host defense to *P. brassicae* and suggests several genes as potential markers for gene expression analysis in other host–*P. brassicae* combinations.

Chitinases, a subgroup of pathogenesis-related proteins, are important plant defense enzymes due to their ability to hydrolyze chitin in pathogen cell walls [47,48]. At2g43570, a putative chitinase gene (*CHI*) from Arabidopsis, was found to be induced by a wide variety of pathogens and elicitors [49]. Its homologs in *B. napus* were also upregulated throughout multiple time-points in resistant and susceptible hosts in response to *P. brassicae* pathotypes 5X and 3A [34,38], and the upregulation of one of these homologs (BnaC03g24270D) was confirmed with the NanoString assay. This gene was also upregulated in a resistant *B. napus* line “ZHE-226” when compared with a susceptible *B. napus* line “10159”, at 0, 12, and 72 h after inoculation with a *P. brassicae* field population [50]. Therefore, *CHI* may also be a promising marker gene for studying host responses to *P. brassicae*.

### 3.3. Advantanges and Limitations of the NanoString nCounter System

In the above sections, we confirmed the robustness of the NanoString nCounter System for gene expression analysis in the study of plant responses to clubroot (Figure 1 and Figure 2 and Table 1). However, why or when should we use this method instead of qPCR or RNA-seq? To answer this question, we compared the three methods in terms of their operational requirements, cost, and time needed for analyses.

**Figure 3:**
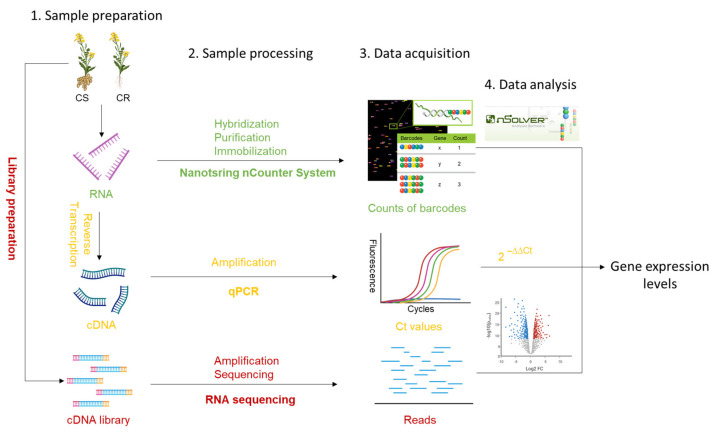
A comparison of the workflows for the NanoString nCounter System, qPCR, and RNA-seq in gene expression analysis of *P. brassicae*/host interactions. Overall, four steps are required to obtain gene expression levels: (1) sample preparation, (2) sample processing, (3) data acquisition, and (4) data analysis. During sample preparation, RNA, cDNA, and cDNA libraries are obtained from clubroot-resistant (CR) and clubroot-susceptible (CS) plants, which are used as input for the NanoString nCounter System, qPCR, and RNA-seq, respectively. During sample processing, three methods are used to generate signals that can be read by corresponding equipment. Amplification of genes, which is required when using qPCR and RNA-seq to increase signals, is not required for the NanoString nCounter System. Instead, RNA is directly tagged with a capture probe and reporter probe (hybridization), creating a unique target–probe complex. Following hybridization, excess probes are removed (purification), leaving only the purified target–probe complexes which are immobilized and aligned on an imaging surface for counting (https://nanostring.com, accessed on 31 October 2022). During data acquisition and analysis, counts of barcodes measured with the NanoString nCounter System are analyzed using nSolver software developed by the company, Ct values obtained from qPCR are analyzed based on the 2^−∆∆Ct^ method to achieve relative quantification of gene expression, and reads generated from RNA-seq are analyzed using diverse bioinformatics pipelines generating semi-quantitative data.

Gene expression analysis using the NanoString nCounter System, qPCR, or RNA-seq requires four general steps: sample preparation, sample processing, data acquisition, and data analysis (Figure 3). Previous studies suggested that the NanoString assay has a greater tolerance for samples with poor-quality RNA than qPCR or RNA-seq [51,52], which could generate reliable data from highly degraded RNA (RNA integrity value, ~2.0) obtained from formalin-fixed paraffin-embedded samples [53]. NanoString Technologies recommends using RNA samples with 260/280 and 260/230 OD ratios close to 2.0 and a DV200 (the percentage of RNA fragments > 200 nucleotides) > 50% for fresh, frozen, and formalin-fixed paraffin-embedded samples; however, samples with greater fragmentation may still be used as input for the NanoString nCounter System with increased amounts of RNA (https://nanostring.com, accessed on 31 October 2022). Furthermore, because NanoString technology uses RNA as the direct input and detects the fluorescence signals of probes bound to target genes without amplifying those genes [27], it does not require cDNA synthesis or the amplification steps needed for qPCR and RNA-seq. This not only simplifies operational procedures but also enhances the accuracy of gene quantification because no errors can be introduced in these intermediate steps. In addition, because the supplier streamlines the design of the NanoString probes, experimental times are shortened and there is enhanced consistency with potential future assays. As this was the first study applying the NanoString platform to host gene expression in response to clubroot, we spent some time communicating with the supplier to confirm the probe design. However, it is possible that gene panels can be developed using marker genes for specific pathways, and probes for these genes can be applied to study gene expression in other host–pathogen combinations. In fact, diverse nCounter panels for human research such as cardiovascular disease, infectious diseases, immunology, neuroscience, and oncology are available (https://nanostring.com, accessed on 31 October 2022) and widely applied [54,55,56,57]. A similar system could be established for clubroot studies. In addition, sample processing with the NanoString nCounter System was much faster than with qPCR and RNA-seq (Table 2), and data quantification is performed by a centralized service at our institution. Unlike RNA-seq, which generates large datasets that require bioinformatics expertise and several weeks to analyze, the user-friendly software nSolver made analysis of NanoString-generated data fast and simple (less than one day). Furthermore, we quantified almost three times the number of genes at only twice the cost when using the NanoString nCounter System vs. qPCR (Table 2).

While the NanoString nCounter System shows promise for gene expression studies with clubroot, it does have several limitations that need to be taken into account when considering this technology for some applications. First, like qPCR, the NanoString platform is restricted to a limited number of genes, and prior knowledge of the target genes is required for the probe design. As a result, unlike in RNA-seq, novel expressed transcripts or genes that could be important but not selected would not be identified. In addition, reference (housekeeping) genes with stable expression across studied samples and conditions are required for NanoString analysis, just as they are needed for qPCR. The stability of these reference genes should be validated prior to their use, but running NanoString assays for the sole purpose of reference gene validation could be costly. As such, it may be desirable to select reference genes that have been used in similar studies and include at least three reference genes along with the target genes as suggested by the manufacturer (https://nanostring.com, accessed on 31 October 2022). Lastly, the probe design for the NanoString nCounter System requires no more than 85% sequence homology between target and non-target sequences to avoid the binding of probes to non-specific targets (https://nanostring.com, accessed on 31 October 2022). This could be a challenge for *Brassica* species that have multiple homolog genes due to whole genome duplication, including the allotetraploid *B. napus* that is derived from diploid *B. rapa* and *B. oleracea* progenitors [58]. This is a challenge not only with the NanoString assay but also with qPCR.

To date, RNA-seq and qPCR are the most cost-effective technologies for genome-wide and small-scale gene expression studies, respectively. However, the NanoString nCounter System may be more cost-effective and efficient than either of these two methods for screening the expression of a few dozen to hundreds of genes. For the validation of RNA-seq data, the cost of using the NanoString is comparable to qPCR, but the former may provide improved robustness, operational simplicity, and efficiency from sample preparation to data analysis.

## 4. Materials and Methods

### 4.1. Plant Inoculation, Sample Collection, and RNA Extraction

The RNA used in the current study was obtained from a previous analysis of the transcriptomic response of *B. napus* to *P. brassicae* [34]. The experimental design was described previously [34]. Briefly, a *P. brassicae* inoculum suspension was prepared with resting spores extracted from *B. napus* root galls infected with a field isolate representing pathotype 3A, as classified on the CCD set [10], and adjusted to a final concentration of 1 × 10^7^ resting spores/mL with distilled water. Two rutabaga cultivars “Wilhelmsburger” and “Laurentian”, which are resistant and susceptible to pathotype 3A, respectively, were used as hosts. Plants were grown under long days (16 h light/8 h dark) at 22 °C, and inoculated roots were harvested at 7, 14, and 21 days after inoculation (dai). Controls were mock-inoculated with water. Each treatment consisted of five independent biological replicates, with 27 pooled plants in each replicate. RNA was isolated from 0.1 mL of homogenized root tissue harvested from each treatment with a trizol–chloroform–isopropanol extraction protocol, followed by a cleanup step using a RNeasy Mini Kit (Qiagen, Hilden, Germany) and a DNA removal step using DNAse (Qiagen, Hilden, Germany).

### 4.2. Gene Expression Analysis by the NanoString nCounter System

Probe sequences for the target genes were designed and synthesized following a standard procedure [59]. A list of 33 genes, including 29 target genes and 4 reference genes, was submitted to NanoString Technologies, with the company generating a report containing information on the reporter and capture probe pairs for each gene. This report was reviewed by the authors of this study to confirm probe specificity and quality, and once approved, a CodeSet containing the reporter and capture probe pairs was manufactured by NanoString Technologies. The target and reporter probe CodeSets for the 33 genes are provided in Table S1.

Four of five replicates of each RNA sample, including three that had been used for RNA-seq [34], were subjected to gene quantification with the NanoString nCounter System. To quantify gene expression levels, 5 μL of a 30 ng/μL RNA suspension was first subjected to a hybridization step in a C1000 Touch Thermal Cycler (Bio-Rad, Hercules, CA, USA) according to the nCounter XT CodeSet Gene Expression Assays Protocol (NanoString Technologies). Briefly, samples were hybridized at 65 °C for 18 h in a 15 μL volume consisting of 3 μL of Reporter CodeSet (NanoString Technologies), 5 μL of hybridization buffer (NanoString Technologies), 5 μL RNA sample, and 2 μL capture probeset (NanoString Technologies). After hybridization, the samples were placed in a NanoString nCounter FLEX Analysis System (NanoString Technologies) as per the manufacturer’s recommendations, which included purification and immobilization in a Prep Station (NanoString Technologies) (2–3 h) and data collection in a Digital Analyzer (NanoString Technologies). The nSolver Analysis Software 4.0 (NanoString Technologies) was used for quality control assessment and data normalization, as per the manufacturer’s recommendations. Expression levels of target genes were normalized to positive and negative controls and reference genes. Positive and negative controls were manufactured with the nCounter Gene Expression Assay (NanoString Technologies) to control noise caused by the platform; positive controls also adjust variations that exist across samples within and among different batches loaded to the platform (https://nanostring.com, accessed on 31 October 2022). The positive controls, which consisted of six synthetic DNA control targets, were used to measure the efficiency of the hybridization reaction and to check the linearity performance of the assay. Negative controls, which were eight negative control probes that should not hybridize to any targets within the sample, were used to set the background thresholds. The reference genes used in this study included *GDI1*, *TIP41*, *AP2M*, and *TUA5* (Supplementary Table S1), which had been used to normalize expression levels of target genes in qPCR when studying the host response to *P. brassicae* [34,38]. The stability of the four reference genes was confirmed by evaluating the count data that had been normalized by positive controls. Pearson correlations of the log_2_(count) values of the reference genes between each sample were calculated using the “cor()” function and plotted into a heatmap with the packages “reshape2” [60] and “ggplot2” [61] in RStudio. In addition, the stability of the reference genes was analyzed using BestKeeper [35] and compared with the results of Zhou et al. (2020) obtained by qPCR [34]. Fold changes in expression levels of each gene were obtained by normalizing the counts of inoculated samples and non-inoculated controls. The log_2_ transformed fold change value of each gene was compared with the corresponding data generated by RNA-seq and qPCR in the previous transcriptomic study [34].

### 4.3. Correlation Test and Heatmap

We performed a Pearson correlation test between the results from the NanoString nCounter System and RNA-seq data by comparing the log_2_ fold change values of the 29 genes for each cultivar and time-point, using the “ggpubr” package in RStudio [62]. Eight of the 29 genes had also been validated using qPCR by Zhou et al. (2020) [34], so we performed the Pearson correlation test to compare expression changes of these eight genes as measured using the NanoString nCounter System with the data generated using RNA-seq and qPCR. Heatmaps of the log_2_ fold change values of the 29 genes were generated using the “gplots” package in RStudio [63] to visualize and compare expression of individual genes measured using the NanoString nCounter System and RNA-seq.

## 5. Conclusions

The recent application of RNA-seq has generated large amounts of gene expression data, greatly improving the understanding of the molecular mechanisms of pathogenesis and resistance in *P. brassicae*–host interactions. Because of the high-throughput nature of RNA-seq associated with uncertainty in gene expression quantification, it is necessary to validate these data. Additionally, there is an increasing interest in studying the expression of subsets of genes for larger sample sizes, such as different host and *P. brassicae* pathotype combinations, more time-points, and more tissue types. To date, the quantification of the expression of limited numbers of genes is achieved mainly by qPCR, which may be laborious and time-consuming when the sample size is increased. The NanoString nCounter System, which can achieve fast quantification of RNA, has been widely used to study human diseases but is rarely applied to studies with plants or plant pathogens. In this study, we used the NanoString nCounter System to quantify the expression of 29 genes in two rutabaga cultivars in response to *P. brassicae* and confirmed the robustness of this system by comparing the results generated with this technology to data generated by qPCR and RNA-seq. Some of the genes studied may be important for the host response to clubroot and, therefore, could be good candidates for creating NanoString gene panels that can be studied in other host–*P. brassicae* combinations. Lastly, we compared the three technologies in terms of their operational requirements, time, and cost. We believe that the NanoString nCounter System is a robust, sensitive, efficient, and highly reproducible technology for gene expression analysis and may be an alternative to qPCR to validate RNA-seq data or to study the expression of subsets of genes, with potential relevance to the host response to clubroot.

## Figures and Tables

**Table 1:** Correlation of expression of 8 genes based on log2 fold changes, as determined using the NanoString nCounter System vs. qPCR and RNA-seq analyses.

	NanoString vs. RNA-seq		NanoString vs. qPCR	
	*R*	*p*	*R*	*p*
Laurentian_7 dai	0.98	2.73 × 10^−5^	0.96	2.17 × 10^−4^
Laurentian_14 dai	0.99	2.05 × 10^−6^	0.97	6.82 × 10^−5^
Laurentian_21 dai	0.99	5.97 × 10^−7^	0.96	1.30 × 10^−4^
Wilhelmsburger_7 dai	0.91	1.80 × 10^−3^	0.98	2.53 × 10^−5^
Wilhelmsburger_14 dai	0.93	1.30 × 10^−4^	0.94	6.11 × 10^−4^
Wilhelmsburger_21 dai	0.95	2.97 × 10^−4^	0.90	2.11 × 10^−3^

**Table 2:** Comparison of procedures, time, and costs associated with the use of qPCR, the NanoString nCounter System, and RNA-seq for gene expression analysis, based on real samples used in this study and in a previous study [34].

Assays	qPCR	NanoString nCounter System	RNA Sequencing
No. of genes	13 (10 target genes plus 3 reference genes)	33 (29 target genes plus 4 reference genes)	All expressed genes
No. of samples	48 (12 treatments × 4 biological replicates)	48 (12 treatments × 4 biological replicates)	36 (12 treatments × 3 biological replicates)
Activities (Researchers)	Primer design cDNA synthesis qPCR Data analysis	Interact with supplier (NanoString Technologies) Provide RNA samples Confirm specificity of CodeSets Data analysis	Interact with sequencing company Provide RNA samples Confirm sequencing parameters Data analysis
Activities (Supplier/ Service Provider)	None	Design and synthesize CodeSets Provide materials and equipment Service for sample processing	Provide materials and equipment Construct cDNA library Sequencing
Estimated timeline	1 week for cDNA synthesis and primer design 1 month for qPCR (primer efficiency test + stability testing of reference genes + quantifying expression levels for target genes) 1 day for gene expression analysis	1 to 2 months for CodeSets design and synthesis 1 week for delivery and processing of samples 1 day for gene expression analysis	1 week communicating with the sequencing company 1 to 2 months for delivery and processing of samples 1 week to 1 month for gene expression analysis
Cost	~$3000 CAD	~$6000 CAD	~$11,000 CAD

## Data Availability

Data are available from the corresponding author upon reasonable request.

## Supplementary Materials

The following supporting information can be downloaded at: https://www.mdpi.com/article/10.3390/ijms232415581/s1, Table S1: Genes for the NanoString nCounter System; Table S2: Stability of log2(Count) of reference genes obtained with the NanoString nCounter System, based on BestKeeper; Table S3: Stability of Ct values of reference genes obtained via qPCR, based on BestKeeper; Figure S1: Distribution of Pearson correlation values of the log_2_ count of four reference genes between each sample.
