# Supplementary figures and images for: Application of the NanoString nCounter System as an Alternative Method to Investigate Molecular Mechanisms Involved in Host Plant Responses to Plasmodiophora brassicae
Source: Int J Mol Sci. 2022 Dec 8;23(24):15581. doi: 10.3390/ijms232415581 (PMC9779335; doi:10.3390/ijms232415581)

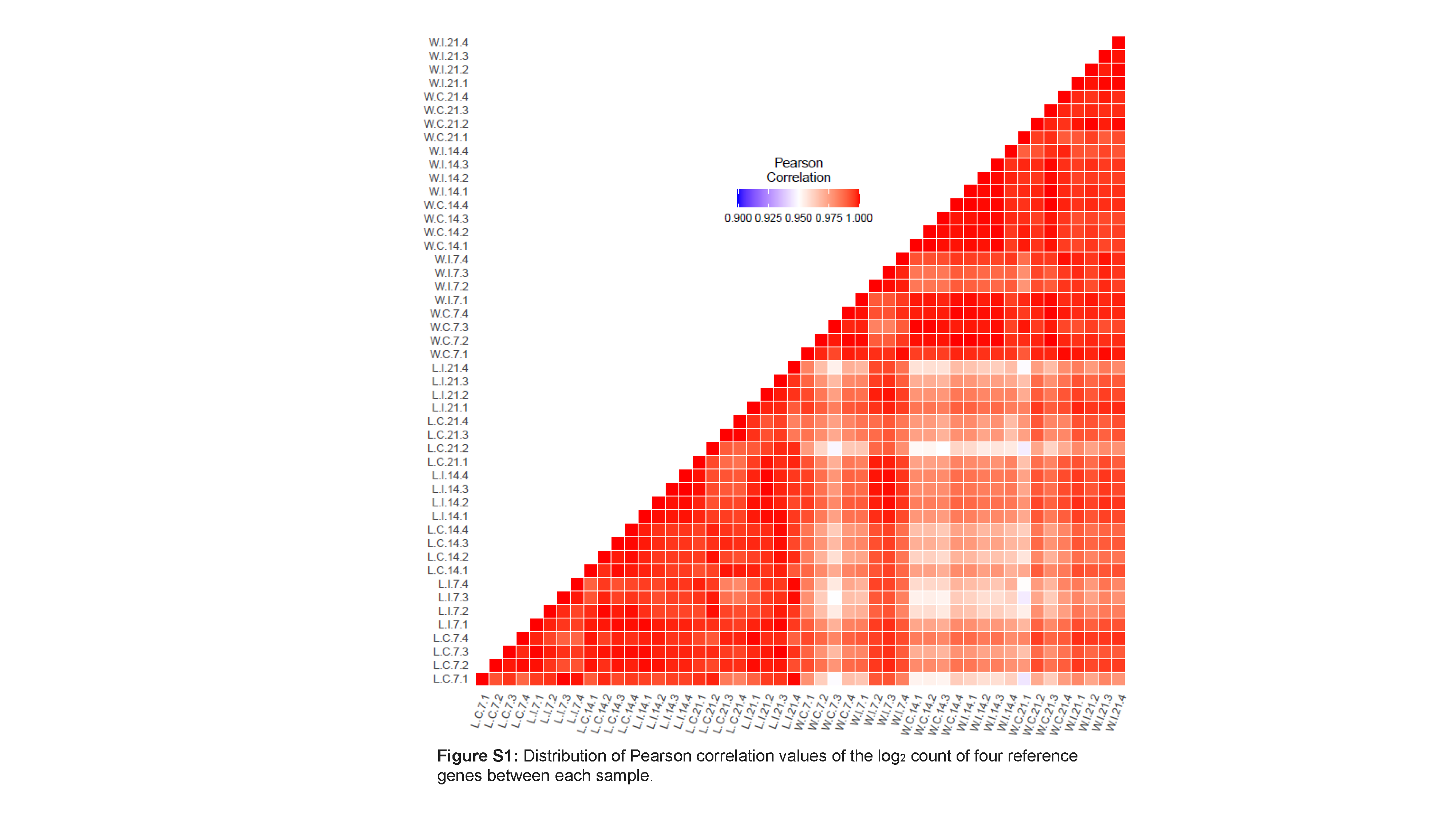

Supplement: Supplementary file 1 [file ijms-23-15581-s001.zip › Figure S1.tiff]
